# “Kambakutaisoto” and Emotional Instability Associated With Premenstrual Syndrome

**DOI:** 10.3389/fnut.2021.760958

**Published:** 2021-10-25

**Authors:** Atsuko Shiota, Chika Shime, Kyoko Nakai, Mitsuru Kageyama

**Affiliations:** ^1^Department of Health Sciences, Faculty of Medicine, Kagawa University, Kitagun, Japan; ^2^Shime Clinic, Kyoto, Japan; ^3^Minamimorimachi Ladies Clinic, Osaka, Japan; ^4^Kageyama Clinic, Osaka, Japan

**Keywords:** premenstrual syndrome (PMS), Japanese Kampo medicine, kambakutaisoto, functional hypoglycemia, tryptophan

## Abstract

Many women suffer from premenstrual syndrome (PMS), which can be considered a modern illness in this busy society; mental symptoms, such as irritability, often affect the surroundings and result in loss of self-confidence. The symptoms of PMS are diverse, and it is often difficult to treat psychiatric and social symptoms with low-dose estrogen progestin combination drug (LEP) alone. Selective serotonin reuptake inhibitors (SSRIs) are also effective; however, many are unable to take them owing to their side effects. “Kambakutaisoto” is a Kampo medicine consisting of “jujube,” “licorice,” and “wheat,” which is often described as “food”; however, it is highly effective in treating emotional instability attributed to PMS in sensitive young women. There are many reports on the effects of kambakutaisoto; the molecular nutritional findings of kambakutaisoto, which has dramatic effects despite its mild composition of crude drugs, have also been reported, suggesting an association with premenstrual exacerbation of functional hypoglycemia. A narrative review of its clinical effects on PMS and the results of molecular nutrition studies was performed.

## Introduction

Many women today suffer from premenstrual syndrome (PMS) (1). PMS can be considered a modern illness associated with a busy lifestyle; mental symptoms such as irritability often affect one's surroundings and results in a loss of self-confidence (2).

The cause of PMS has not yet been elucidated in detail, although there is a theory that serotonergic neurons are highly sensitive to progesterone (3). The symptoms of PMS are diverse, and it is often difficult to treat psychiatric and social symptoms with low-dose estrogen progestin combination drug (LEP) alone (4). Serotonin reuptake inhibitors (SSRIs) are effective; however, many patients are unable to take them owing to their side effects (3). Kampo medicine could potentially be effective in the treatment of PMS if Kampo medicine is selected based on a pattern called “sho” that is specific to Kampo medicine. “Kambakutaisoto” is a Kampo medicine consisting of jujube, licorice, and wheat, which is often described as food; this medicine is highly effective in treating emotional instability attributed to PMS in sensitive young women (5–15).

Why does kambakutaisoto have a dramatic sedative effect even though it is a mild formulation of crude drugs that could be considered to be food? One answer is that, from a molecular nutritional point of view, kambakutaisoto stabilizes blood sugar and contains components involved in the production of serotonin and gamma amino butyric acid (GABA) (10, 16). Is it related to the recently discussed mechanism of functional hypoglycemia? During the luteal phase, functional hypoglycemia could be exacerbated and manifested as a symptom of PMS (17, 18). Kambakutaisoto could be effective in treating hypoglycemia as an underlying cause. Hence, we have reviewed the effects of kambakutaisoto on PMS, the molecular nutritional content of kambakutaisoto, and functional hypoglycemia.

## Diagnosis and Treatment in Kampo Medicine

In Kampo medicine, which is a traditional Japanese medicine, various crude drugs work in conjunction, presenting the effects of mutual relationships among crude drugs. The crude drugs are mainly natural and gentle, such as grassroots bark.

**Figure 1:**
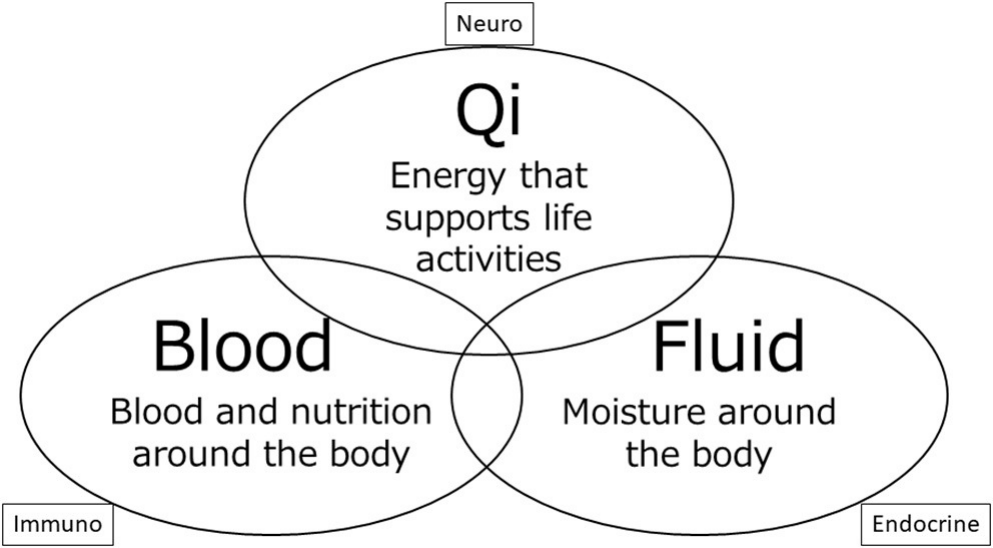
Theory of Qi, Blood, Fluid. The three elements showing in this figure are thought to maintain the living body within the Kampo medicine framework. Three elements go around the mind and body, and make them work. Symptoms are caused by impaired circulation and quantity of three elements.

Kampo medicine has gradually begun to reemerge and now occupies a considerable position in the field of medical practice in Japan. We introduce one of the theories of choosing Kampo medicine in Figure 1.

In Kampo medicine, qi is considered a life energy source that is sourced from food and air. The concept of qi plays a primary role in the assessment of clinical condition in Kampo medicine, where physical disorders indicate abnormal quantities or locations of qi. In the body, part of qi becomes a liquid in order to build and maintain life. Among the liquids, red liquid is blood, and the remaining colorless liquids are fluid. The three entities of qi, blood, and fluid bear a close relation to the neuro-immuno-endocrine triangle familiar to Western medicine. This key concept is the basis for assessing the clinical conditions in Kampo medicine (19).

## PMS and Kampo Medicine

According to the Kampo medical interpretation, the luteal phase is the time when qi and blood flow downward (assuming pregnancy). If there is qi depression or static blood in the base, various symptoms are likely to occur as PMS. Moreover, mental symptoms, such as irritability, often affect the surroundings and result in loss of self-confidence (2). Many Kampo medicines have psychotropic effects. Kamishoyosan is a formulation against static blood and qi depression, and there are many reports on its effective in PMS (20–23). Shiota and Hata examined 38 cases of PMS (5). For those who felt betrayed by their expectations of others and turned their anger toward others, kamishoyosan was an effective treatment. Yokukansankachimpihange was effective for those who felt betrayed by their expectations for themselves and expressed anger toward themselves. Kambakutaisoto was effective for those who were delicate, emotionally unstable, and could not see themselves objectively (5).

## PMS and “Kambakutaisoto”

Since 2005, there have been reports of the use of kambakutaisoto for PMS and premenstrual dysphoric disorder (PMDD) (5). Nakai et al. reported that kambakutaisoto was dramatically effective in 14 cases of PMS (13–15). In many of these reported cases, 2.5–5 g of kambakutaisoto was used as a single dose as needed. In effective cases, it takes <1 h from the time of taking the drug until the effect is realized, and in many cases, the effect is seen within 30 min. Supplementary Table 1 shows 32 cases in which the reported kambakutaisoto formulation was effective. A common feature of the 32 cases was uncontrollable premenstrual emotional instability. According to a review by Nakai et al. (15), which summarizes 14 cases in detail, many symptoms, such as yawning (86%, 12/14), edema (79%, 11/14), insomnia or drowsiness (64%: 9/14), and constipation (64%, 9/14) were reported. Impulsive symptoms, such as overeating (50%, 7/14), were also observed. In other studies, overeating, insomnia, constipation, and fatigue were common. Edema and constipation are findings of fluid retention and static blood, and are common symptoms in premenstrual women; hence, it cannot be assumed to be a characteristic symptom of women presenting the effect of kambakutaisoto. Regarding the findings of the Kampo assessment for 14 cases with abdominal examination, fluids retention in stomach, palpitation in the supra-umbilical region, and para-umbilical tenderness and resistance, were observed in nearly 80% of the cases. There were findings of fluid retention and static blood. Fullness and discomfort in the chest and hypochondrium and abdominal muscle tension, which are thought to be reflective of excessive stress, were also observed in approximately half the cases. In other words, if fluid retention or static blood is found in the abdominal examination, the corresponding Kampo medicine should be taken regularly. If one has impulsive symptoms, such as emotional instability or overeating, effects of kambakutaisoto can be treated with a single dose.

## Kambakutaisoto: Outstanding Cases and Functional Hypoglycemia

Kambakutaisoto is a Kampo medicine consisting of jujube, licorice, and wheat. The details are listed in STORK (http://mpdb.nibiohn.go.jp/kconsort/kconsort.html) (24). The original text describing this is “Jinguiyaolue”: Women's Chronic Miscellaneous Diseases,” which was written 1,800 years ago. According to Classics of traditional medicine, it is effective against hysterical attacks, sadness, crying, and yawning symptoms in women who appear as if they are possessed (25). The insurance coverage for Japanese Kampo medicine manufacturers includes crying at night, neurosis, and insomnia. In addition, it is widely used for urgent, unexplained frustration and excitement as well as convulsions.

We considered the characteristics of crude drugs that make up kambakutaisoto. Licorice has tension-relieving, sedative, analgesic, and stomach-healing effects, and “Yakucho” is said to cure urgency (26). Jujube has the effect of strengthening the digestive tract, stabilizing the mind, and relieving tension, and “Yakucho” is said to stop cramping (27). Although there is little mention of wheat in the literature, the “Bencao Gangmu (Materia Medica)” states that it nourishes the mind and is thought to have the effect of supplementing energy and stabilizing the mind.

All three crude drugs have mild conditions; however, in clinical practice, they show a sharp effect in a short amount of time. So far, it has been argued that the underlying mechanisms include a blood glucose-retaining effect, effects of tryptophan (involved in the production of serotonin) in the oral cavity, and endorphins secretion (10, 15). However, this has not been verified.

The concept of functional hypoglycemia has been advocated since the 1980s (28, 29). This concept was introduced to Japan by Osawa (30) in the 1990s and, along with Kashiwazaki, is actively implemented in treating patients (31, 32). The pathology is thought to be associated with eating a diet with rapidly fluctuating blood glucose levels (a diet with a high glycemic index: a GI diet), which overreacts or disrupts glycemic control. “Hypoglycemia” is generally associated with diabetes treatment. In addition, insulinoma often occurs in “fasting hypoglycemia” and “postprandial hypoglycemia (reactive hypoglycemia)” often occurs in the so-called dumping syndrome following gastric surgery and in the early stages of type 2 diabetes. However, hypoglycemia could occur even in people without such a background; hypoglycemia has been confirmed by oral precision glucose tolerance test (OGTT), and many cases have been reported in which improvement of dietary habits, such as sugar restriction, was effective (33–35).

**Figure 2:**
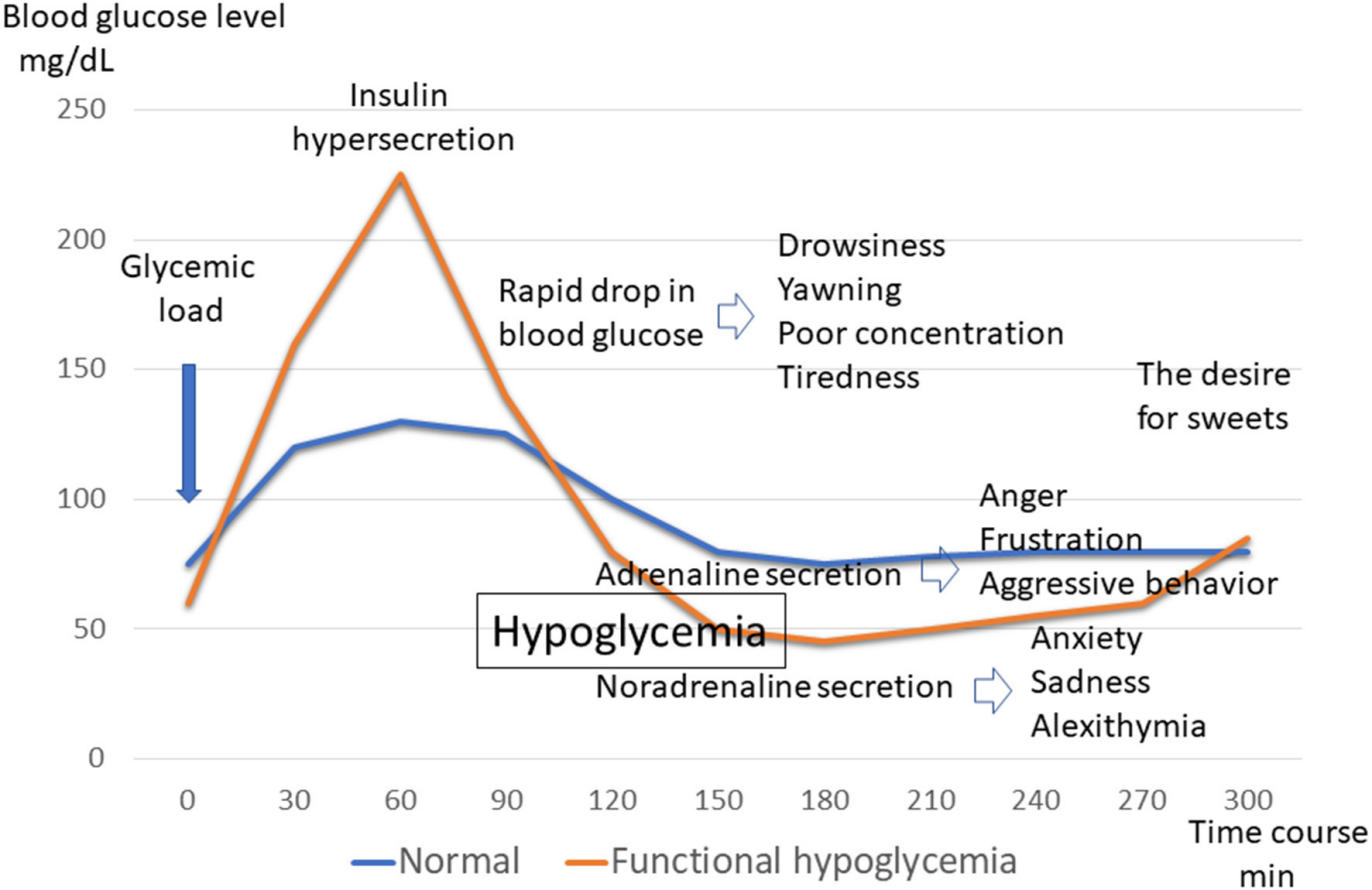
Blood glucose levels and symptoms of functional hypoglycemia. Glycemic fluctuations in normal and functional hypoglycemic patterns following glucose loading. Changes in symptoms associated with glycemic fluctuations in functional hypoglycemia.

When the blood sugar level rises sharply owing to a high GI diet, a large amount of insulin is secreted to regulate it in an attempt to lower the blood sugar level. When trying to raise the blood sugar levels that have dropped too low, hormones such as adrenaline, noradrenaline, and cortisol are also rapidly secreted. In other words, after an uplifting mood due to hyperglycemia, a rapid decrease in blood glucose levels occurs owing to excessive insulin secretion. Following this, drowsiness, yawning, poor concentration, and tiredness appear. Next, anger, frustration, and aggressive behavior owing to the adrenaline secreted to raise blood sugar appears. Similarly, anxiety, sadness, and alexithymia owing to noradrenaline secretion are observed. This causes an urge to eat sweets, and the cycle is repeated again (16) (Figure 2). It is very surprising that these symptoms are consistent with the rules of kambakutaisoto and the symptoms that apply to prominent cases.

Several studies have been conducted on the relationship between the female menstrual cycle and blood glucose. During the follicular phase, blood glucose tends to be low due to increased insulin sensitivity and decreased insulin resistance owing to the effects of estrogen. It is said that during the luteal phase, insulin sensitivity decreases and insulin resistance increases owing to the effects of progesterone, resulting in an increase in blood glucose (18).

It has also been reported that 70% of women with type 1 diabetes had elevated blood glucose before menstruation and half of them decreased on the first day of menstruation (36).

Among PMS, those with strong mental symptoms are called Premenstrual Dysphoric Disorder (PMDD), and the diagnostic criteria include the item of “overeating.” Even women who cannot be diagnosed with PMDD, PMS often feel increased appetite. If blood glucose tends to be high during the luteal phase, why is appetite increased?

It has been reported that during the luteal phase, progesterone acts as a mechanism for mobilizing glucose and accumulating fat, promoting hunger and increased appetite (37). Leptin levels have been reported to be associated with overeating in women with normal weight PMDD (38). In addition, since PMS symptoms are more likely to occur in women who are highly sensitive to progesterone in serotonergic neurons, it is possible that a decrease in serotonin increases appetite. There is also a report that after absorption of the sweet solution, serotonin in the brain increased and the mood improved (39). Appetite is also increased as a simple method of coping with stress (40). When overeating occurs in the luteal phase in this way, reactive hypoglycemia is likely to occur because of impaired glucose tolerance following after decreased insulin sensitivity, compared to the follicular phase. This is similar to reactive hypoglycemia in the early stages of type 2 diabetes. It is conceivable that those who have “functional hypoglycemia” as a base, the condition may worsen during the luteal phase; as a result, symptoms may become apparent.

There are studies examining blood glucose in patients with PMS and the control group (41). According to the results, the blood glucose level in the luteal phase was significantly lower than that in the control group. However, since this study is a one-point test of blood sampling results, it may not be an accurate representation of the subject's postprandial hypoglycemic status.

## Molecular Nutritional Considerations

Is “Kambakutaisoto” really effective in improving the symptoms caused by functional hypoglycemia? To support this, we would like to introduce the molecular nutritional consideration of kambakutaisoto, as clarified by Shime et al. (16).

These researchers asked the Japan Food Research Laboratories to measure 55 items, such as proteins, lipids, electrolytes, carbohydrates, sugar, dietary fiber, energy, vitamins, and amino acids (Supplementary Table 2) (100 g), sugar (84.8 g; fructose, 8.85 g; glucose, 7.87 g; sucrose, 2.51 g; maltose, 0.21 g; lactose, 51.5 g), dietary fiber (3.8 g), protein (3.7 g), lipids (1.7 g), iron, calcium, potassium, magnesium, copper, zinc, manganese, vitamins B1, B2, B6, B12, and K, folic acid, pantothenic acid, biotin, and niacin, 18 amino acids, tryptophan (25 mg), and glutamic acid (342 mg) were observed. Based on these results, the following is suggested.

1. Monosaccharides and disaccharides are confirmed as sugars, and dietary fiber is also included to stabilize blood glucose disorders attributed to functional hypoglycemia.
2. This mixture contains tryptophan, minerals, and vitamins for conversion to serotonin and melatonin. It also contains glutamic acid, which promotes GABA production.
3. Various nutrients related to these systems can be ingested at the same time and act as a complex system.

The basic treatment for functional hypoglycemia is improvement of eating habits; however, it is not cured in a short period of time; it often takes several years or more to improve symptoms. If the symptoms worsen, they can be alleviated by taking a small amount of low GI diet every few hours, though we consider kambakutaisoto effective during such times.

Nakajima describes the relationship between kambakutaisoto and intestinal flora as follows (42): wheat contains all the ingredients necessary for tryptophan metabolism by the intestinal flora. However, its metabolism is directed toward kynurenine production, which causes mental disorders, rather than serotonin production under chronic inflammation and stress. Licorice exerts corticosteroid action through the intestinal flora. Due to its anti-inflammatory effect, tryptophan metabolism lowers kynurenine and leads to serotonin production.

Indeed, the effects of Kampo medicine are complex, and the relationship between the nutritional aspects of crude drugs and the intestinal flora is also interesting.

## Kampo Medicine: A Multi-component System

**Figure 3:**
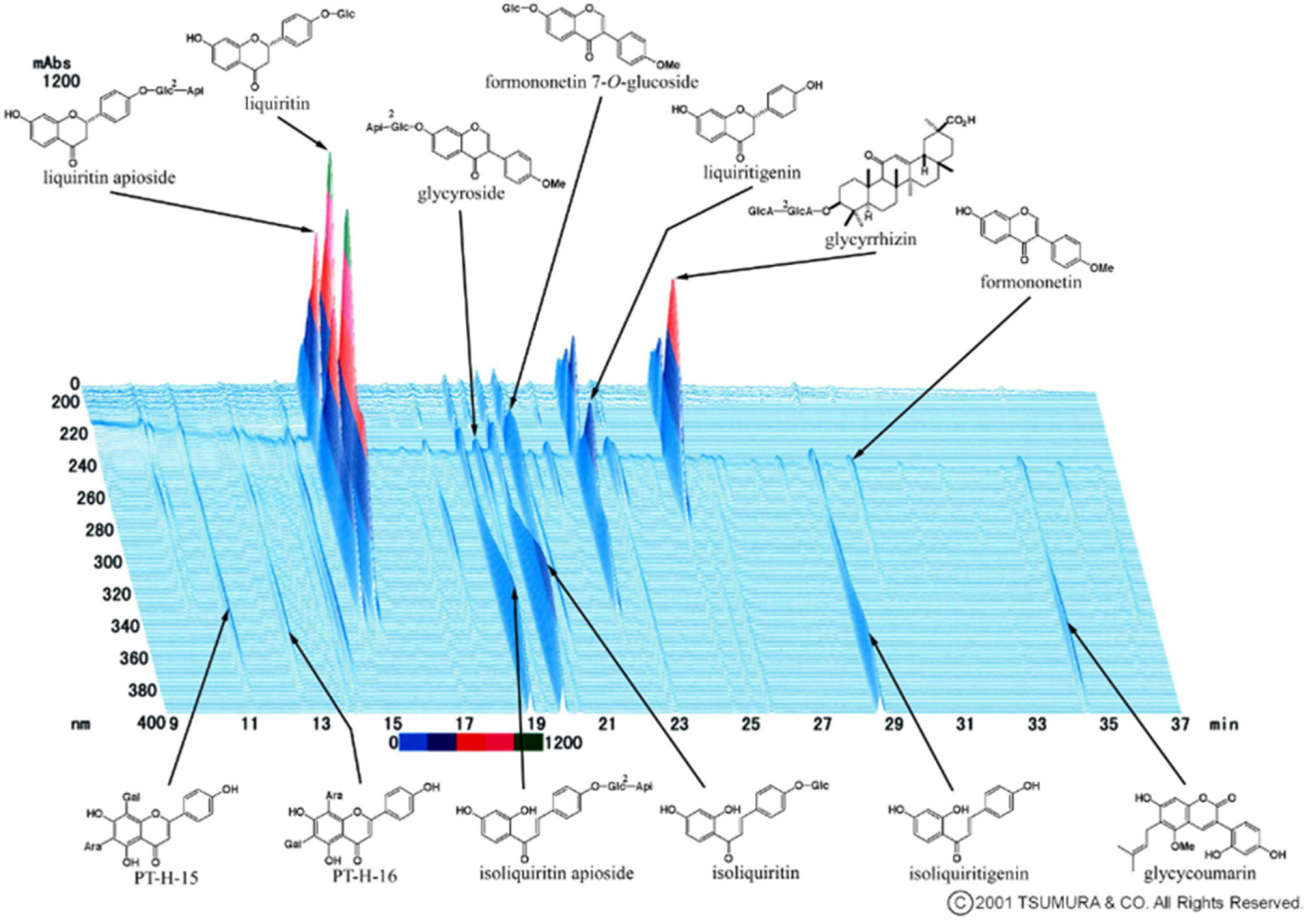
3D HPLC Pattern of Kambakutaisoto. 3D-HPLC (High Performance Liquid Chromatography) fingerprint evaluation of Kambakutaisoto. Using this method, the equivalence of herbal preparations consisting of botanical crude drugs is evaluated.

Kampo medicine is composed of multiple botanical ingredients, including many pharmacologically active substances. For quality control, it is important to make efforts to stabilize the quality of raw materials at the crude drug level. The 3D-HPLC (High Performance Liquid Chromatography) fingerprint evaluation method is useful for quality control of crude drugs and final products (43). This is one of the methods proposed by the Food and Drug Administration (FDA) and the European Agency for the Evaluation of Medicinal Products (EMEA) for the quality control of botanical drugs. The 3D-HPLC pattern of kambakutaisoto is shown in Figure 3.

Because Kampo medicine are multi-component, they have multiple targets. It is thought that these multi-ingredients of kambakutaisoto have a mild effect on blood glucose regulation and serotonin production from tryptophan, similar to an orchestra.

## Conclusions

We have reviewed the clinical effects of the Japanese Kampo medicine kambakutaisoto on emotional instability associated with PMS, with “functional hypoglycemia” as a keyword, and added molecular nutritional considerations.

We hypothesized that some patients with PMS have symptoms of exacerbation of functional hypoglycemia during the luteal phase, and the characteristic symptoms are uncontrollable emotional instability and urgency. Kambakutaisoto, with molecular nutritional evidence, is very effective for such symptoms.

However, few studies have considered functional hypoglycemia in detail (33–35). There are fewer papers on PMS and glycemic fluctuations (41).

An accurate diagnosis of functional hypoglycemia requires a 5 h OGTT (31, 32).

To the best of our knowledge, there are no PMS research papers investigating hypoglycemia with a 5 h OGTT.

We believe that it is necessary to investigate blood glucose fluctuations in patients with PMS. Currently, there is an excellent glucose monitoring system that can be worn subcutaneously to measure diurnal variations in blood glucose levels in patients with diabetes. It would also be very meaningful to use it to investigate diurnal fluctuations in blood glucose levels in patients with PMS.

We wish to study the fluctuations in blood glucose among patients with PMS as well as blood glucose levels after taking kambakutaisoto. We hope that this review will lead to further research in this field.

## Author's Note

Kambakutaisoto extract granules for ethical use used in Supplementary Tables 1, 2 and Supplementary Figure 3 was made by Tsumura Co., ltd.

## Author Contributions

AS, CS, and KN contributed to conception and design of the study. AS and KN organized the database. CS made a molecular nutritional consideration. AS wrote the first draft of the manuscript. AS, CS, KN, and MK wrote sections of the manuscript. MK supervised the entire treatise. All authors contributed to manuscript revision, read, and approved the submitted version.

## Conflict of Interest

The authors declare that the research was conducted in the absence of any commercial or financial relationships that could be construed as a potential conflict of interest.

## Publisher's Note

All claims expressed in this article are solely those of the authors and do not necessarily represent those of their affiliated organizations, or those of the publisher, the editors and the reviewers. Any product that may be evaluated in this article, or claim that may be made by its manufacturer, is not guaranteed or endorsed by the publisher.
