# Supplementary material for: “Kambakutaisoto” and Emotional Instability Associated With Premenstrual Syndrome
Source: Front Nutr. 2021 Oct 25;8:760958. doi: 10.3389/fnut.2021.760958 (PMC8573044; doi:10.3389/fnut.2021.760958)
Supplement: Supplementary file 1 [file Table_1.DOCX]

Table 1. Symptoms and Findings of Kambakutaisoto in 32 Cases
